# Diversity of ABBA Prenyltransferases in Marine *Streptomyces* sp. CNQ-509: Promiscuous Enzymes for the Biosynthesis of Mixed Terpenoid Compounds

**DOI:** 10.1371/journal.pone.0143237

**Published:** 2015-12-14

**Authors:** Franziska Leipoldt, Philipp Zeyhle, Andreas Kulik, Jörn Kalinowski, Lutz Heide, Leonard Kaysser

**Affiliations:** 1 Pharmaceutical Biology, Pharmaceutical Institute, Eberhard Karls University Tübingen, Tübingen, Germany; 2 German Centre for Infection Research (DZIF), Partner Site Tübingen, Tübingen, Germany; 3 Microbial Biotechnology, Interfaculty Institute of Microbiology and Infection Medicine, Eberhard Karls University Tübingen, Tübingen, Germany; 4 Microbial Genomics and Biotechnology, Center for Biotechnology, Bielefeld University, Bielefeld, Germany; University of New South Wales, AUSTRALIA

## Abstract

Terpenoids are arguably the largest and most diverse family of natural products, featuring prominently in e.g. signalling, self-defence, UV-protection and electron transfer. Prenyltransferases are essential players in terpenoid and hybrid isoprenoid biosynthesis that install isoprene units on target molecules and thereby often modulate their bioactivity. In our search for new prenyltransferase biocatalysts we focused on the marine-derived *Streptomyces* sp. CNQ-509, a particularly rich source of meroterpenoid chemistry. Sequencing and analysis of the genome of *Streptomyces* sp. CNQ-509 revealed seven putative phenol/phenazine-specific ABBA prenyltransferases, and one putative indole-specific ABBA prenyltransferase. To elucidate the substrate specificity of the ABBA prenyltransferases and to learn about their role in secondary metabolism, CnqP1 –CnqP8 were produced in *Escherichia coli* and incubated with various aromatic and isoprenoid substrates. Five of the eight prenyltransferases displayed enzymatic activity. The efficient conversion of dihydroxynaphthalene derivatives by CnqP3 (encoded by *AA958_24325*) and the co-location of *AA958_24325* with genes characteristic for the biosynthesis of THN (tetrahydroxynaphthalene)-derived natural products indicates that the enzyme is involved in the formation of debromomarinone or other naphthoquinone-derived meroterpenoids. Moreover, CnqP3 showed high flexibility towards a range of aromatic and isoprenoid substrates and thus represents an interesting new tool for biocatalytic applications.

## Introduction

Aromatic prenylation reactions are an important step in the biosynthesis of natural products in both primary and secondary metabolism. Particularly the latter leads to an astonishing diversity of chemical structures in plants, fungi and bacteria [1,2]. A new superfamily of aromatic prenyltransferases, the ABBA prenyltransferases, has been a focus of research in recent years. Those enzymes share a characteristic protein fold with a central barrel (PT barrel) consisting of ten anti-parallel β-strands surrounded by α-helices [3,4]. Because the array of the secondary structures is a repeated α-β-β-α motive the term ABBA prenyltransferases has been introduced in literature [5]. ABBA prenyltransferases have been found exclusively in the secondary metabolism and can be divided into two discrete families based on their substrate specificity [4]. One family, the phenol / phenazine prenyltransferases, is mainly found in bacteria and catalyses the prenylation of naphthoquinones [3,6], hydroxybenzoates [7], phenazines [8] or benzodiazepines [9]. The other family is constituted by indole prenyltransferases from bacteria [10,11] and fungi [12].

The majority of ABBA prenyltransferases catalyses a *C*-prenylation by electrophilic aromatic substitution similar to Friedel-Crafts alkylation [13]. Commonly C-1 of an isoprenyl diphosphate is attached to an aromatic scaffold but occasionally a prenylation via C-3 is observed and termed “reverse” prenylation [6]. So far, most biochemically characterised ABBA prenyltransferases were found to be independent of Mg^2+^ ions or other divalent cations lacking a DDxxD motive characteristic for membrane-bound aromatic prenyltransferases of lipoquinone biosynthesis. Moreover, ABBA prenyltransferases share promiscuity for different aromatic substrates [14].

Isoprenoid moieties are important constituents for the immense structural diversity of secondary metabolites and often are crucial for their bioactivity [5,15]. However, the stereo- and regioselective chemical synthesis of prenylated compounds is difficult, costly, and often requires harsh conditions [16,17]. The chemoenzymatic production of terpenoid molecules using soluble and highly promiscuous ABBA prenyltransferase biocatalysts is thus an interesting alternative [4,18].

*Streptomyces* sp. CNQ-509, isolated from a near-shore marine sediment of La Jolla, CA in 2001 [19], has been assigned to the “MAR4” clade [20], a lineage of 57 actinomycetes originating from marine sources. Those strains are considered highly gifted with respect to their secondary metabolites. Particularly notable is the production of diverse mixed terpenoid structures by these bacteria often decorated with halogens e.g. the napyradiomycins [21], azamerone [22] and marinone [23]. The potent biological activities of hybrid isoprenoids makes the MAR4 clade highly interesting for pharmaceutical discovery.

**Fig 1:**
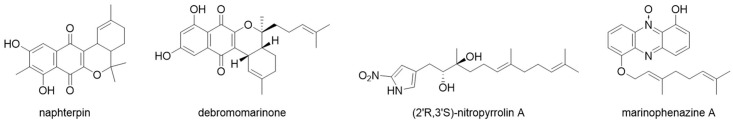
Mixed-terpenoid secondary metabolites of *Streptomyces* sp. CNQ-509.

Within this marine lineage, the terpenoid chemistry of *Streptomyces* sp. CNQ-509 is especially intriguing. In addition to the already known compounds naphterpin [24,25] and debromomarinone [23,26], rare nitropyrrolins [27] and marinophenazines [28] have been isolated from this strain (Fig 1). We speculated that this extraordinary terpenome of *Streptomyces* sp. CNQ-509 correlates to a similarly diverse prenyltransferase biochemistry. To gain deeper insights into the biosynthetic capacity we sequenced the genome of *Streptomyces* sp. CNQ-509 and identified eight putative genes coding for ABBA prenyltransferases [29]. Seven of the predicted enzymes were similar to the phenol / phenazine family (CnqP1-CnqP7) and one was assigned to the (bacterial) indole prenyltransferases (CnqP8). The seven putative phenol / phenazine prenyltransferases were purified and incubated with different isoprenoid and aromatic substrates. Five of the proteins indeed displayed prenyltransferase activity. Of these, CnqP3 is suggested to take part in debromomarinone biosynthesis in the wild-type strain and showed remarkable substrate flexibility.

## Materials and Methods

### Chemicals

Dimethylallyl diphosphate, geranylallyl diphosphate and farnesyl diphosphate were synthesised following the description of Woodside *et al*. [30]. The preparation of flaviolin was conducted according to protocol of Gross *et al*. [31]. Generation of 5,10-dihydrophenazine-1-carboxylate followed the established procedure of Saleh *et al*. [8]. Preparation of 1,6-dihydroxyphenazine was achieved as described by Zeyhle *et al*. [32]. Additional chemicals and molecular biological agents were purchased from standard commercial sources.

### Bacterial strains and culture conditions

*Streptomyces* sp. CNQ-509 was kindly provided by W. Fenical and P. R. Jensen (Scripps Institution of Oceanography, University of California San Diego, USA) and grown as described previously [32]. For cloning experiments *Escherichia coli* XL1 Blue MRF’ (Stratagene) was used and cultured in liquid or on solid Luria-Bertani (LB) medium at 37°C. Selection of recombinant strains was achieved by the addition of kanamycin (50 μg mL^-1^) and tetracycline (12.5 μg mL^-1^) to the medium.

### Genetic procedures, genome sequencing and sequence analysis

DNA isolation and manipulation followed standard methods [33,34]. DNA fragments were isolated from agarose gels by the use of a commercial kit (peqGOLD Gel Extraction Kit). Genomic DNA for cloning via PCR was isolated by lysozyme treatment and phenol / chloroform extraction [33]. A Genome Sequencer FLX System and Titanium chemistry (Roche Applied Science) as well as an Illumina MiSeq were employed to sequence the genomic DNA of *Streptomyces* sp. CNQ-509 [29]. For this purpose paired-end and a whole-genome shot-gun libraries, were generated following standard protocols (Roche Applied Science and Illumina). The resulting sequence reads were assembled with the Newbler software (version 2.8).

The draft genome sequence was analysed using Artemis [35]. For local BLAST searches in the genome BioEdit was employed (http://www.mbio.ncsu.edu/BioEdit/). Database searches were performed with BLAST [36]. Sequences were aligned with ClustalX [37]. Phyre^2^ was used to predict secondary structures [38]. To combine sequence similarities and secondary structure information from aligned sequences, ESPript was utilised [39]. Secondary metabolite gene clusters were identified by antiSMASH 2.0 [40].

### Overexpression and purification of CnqP1 –CnqP7

Genes were amplified from genomic DNA of *Streptomyces* sp. CNQ-509 using the following primers: For *AA958_30735* (CnqP1) AA958_30735_f_pHis8_BamHI (5’-CG**G GAT CC**A TGC CCG AGG CCA CCA AGC-3’) and AA958_30735_r_pHis8_EcoRI (5’-CG**G AAT TC**T CAG TCC GCG GGG CGT ACG C-3’), for *AA958_18620* (CnqP2) AA958_18620_f_pHis8_BamHI (5’-CGC **GGA TCC** ATG TCC GGG GCT AAC GAC G-3’) and AA958_18620_r_pHis8_EcoRI (5’-CG**G AAT TC**T CAG TCC AGG GCC TCG AAC GCC-3’), for *AA958_24325* (CnqP3) AA958_24325_f_pHis8_BamHI (5’-CG**G GAT CC**A TGT CCG GTG CTG CTG ACG-3’) and AA958_24325_r_pHis8_EcoRI (5’-CG**G AAT TC**C TAG TCG TTG AGC GAG TCG AAC G-3’), for *AA958_24270* (CnqP4) AA958_24270_f_pHis8_NcoI (5’-CAT G**CC ATG G**CA TGT CCG GAG CTG TCG-3’) and AA958_24270_r_pHis8_XhoI (5’-CCG **CTC GAG** GCT ACC GCT TGT GCG TCT CC-3’), for *AA958_12625* (CnqP5) AA958_12625_f_pHis8_BamHI (5’-CG**G GAT CC**T TGG CAG ACA AGA CCG AAG T-3’) and AA958_12625_r_pHis8_XhoI (5’-CCG **CTC GAG** TCA GTC GCC CCT GGT GAG-3’), for *AA958_12635* (CnqP6) AA958_12635_f_pHis8_BamHI (5’-CG**G GAT CC**A TGT CCA GTC CCG CAG AGA ACC-3’) and AA958_12635_r_pHis8_EcoRI (5’-CG**G AAT TC**A CTA GCC GGA GGC GGG GGG-3’) and for *AA958_12645* (CnqP7) AA958_12645_f_pHis8_BamHI (5’-CG**G GAT CC**A ATG AGC ACC AAT CCT GGG ATC G-3’) and AA958_12645_r_pHis8_EcoRI (5’-CG**G AAT TC**T CAG CTC CCG CGG CGT ACC-3’). The bold letters represent the restriction sites. According to manual BLAST search and comparison with biochemically characterised homologues, the following genes were extended on the 5’-end: *AA958_30735* (60 bp), *AA958_18620* (39 bp) and *AA958_12645* (87 bp). Amplified PCR fragments were digested with the indicated restriction endonucleases and ligated into the same sites of pHis_8_[41]. Obtained plasmids pFL34, pFL35, pFL36, pFL37, pFL38, pFL39 and pFL40 were confirmed by restriction mapping and sequencing.

For protein overproduction, 1 L of Terrific Broth medium (kanamycin 50 μg mL^-1^, chloramphenicol 25 μg mL^-1^) was inoculated with 37.5 mL of an overnight culture of *Escherichia coli* Rosetta2(DE3)pLysS (Novagene) harbouring the respective expression plasmids. The culture was grown at 37°C and 250 rpm to an OD_600_ of 0.6, then the cultivation temperature was adjusted to 20°C and isopropyl β-d-1-thiogalactopyranoside was added to a final concentration of 0.5 mM. After 6–7 h of cultivation the cells were harvested by centrifugation (2,700 × g, 10 min, 4°C). The obtained pellet was resuspended in 2.5 mL of a lysis buffer (50 mM Tris-HCl, pH 8.0, 500 mM NaCl, 10% (v/v) glycerol, 20 mM imidazole, 10 mM β-mercaptoethanol, 1% Tween 20, 0.5 mg mL^-1^ lysozyme, 0.5 mM phenylmethanesulfonyl fluoride) per 1 g cells and stirred at 4°C for 30 min. The slurry was treated with a Branson sonifier W-250 D at 4°C to rupture the cells. To separate soluble and insoluble components, the lysate was centrifuged (38,720 × g, 45 min, 4°C). Purification of the supernatant was carried out by the utilisation of a 1.5 mL nickel-nitrilotriacetic acid-agarose (Ni-NTA) resin column (GE Healthcare) according to the manufacturer’s instructions. A linear gradient of 20 to 250 mM imidazole (in 50 mM Tris-HCl pH 8.0, 500 mM NaCl, 10% (v/v) glycerol, 10 mM β-mercaptoethanol) within 50 min was applied for elution. The solution was passed through PD-10 columns (GE Healthcare) to exchange the buffer and eluted with 10 mM Tris-HCl pH 8.0, 10% (v/v) glycerol and 2 mM 1,4-dithiothreitol.

### Assay for prenyltransferase activity

The reaction mixtures (100 μL) contained 50 mM Tris-HCl pH 8.75, 5 mM MgCl_2_, 10 mM ascorbic acid, 200 mM NaCl, 1 mM isoprenoid substrate, 2 mM aromatic substrate and 10 μM enzyme (CnqP5 5 μM). We previously showed that most ABBA prenyltransferases are active under these conditions. In case of 5,10-dihydrophenazine-1-carboxylate, the substrate was freshly prepared as described previously and oxidized to phenazine-1-carboxylate before extraction [7]. After incubation for 2 h at 30°C, the assays were put on ice and extracted with 100 μL ethyl acetate containing 2.5% (v/v) formic acid. The mixtures were vortexed and centrifuged and the organic layer (80 μL) was collected for evaporation. The residues were dissolved in 100 μL methanol for the analysis via high-performance liquid chromatography (HPLC). For the substrate 2,7-DHN, acidification and extraction with ethyl acetate were replaced by direct addition of 100 μL methanol to the assay to precipitate the enzyme. After brief storage at -75°C and centrifugation the supernatant was directly analysed by reversed phase liquid chromatography. HPLC was carried out using a Reprospher 100 C 18-DE column (4.6 × 150 mm, 5 μm; Dr. Maisch GmbH, Ammerbuch) at a flow rate of 1 mL min^-1^, 40°C and a linear gradient from 40 to 100% solvent B in 12 min and additional 1 min at 100% solvent B (solvent A: water with 0.1% formic acid (v/v), solvent B: methanol with 0.1% formic acid (v/v)). Absorbance was measured at 288 nm (flaviolin), 260 nm (genistein), 286 nm (1,3-DHN, 1,6-DHN, 2,7-DHN), 260 nm (phenazine-1-carboxylic acid) and 350 nm (2-nitropyrrole). Furthermore, a photodiode array detector (DAD) recorded the UV spectrum from 200 to 400 nm. To generate reference compounds, the ABBA prenyltransferases Fnq26 and NphB were used and treated in the same way as the assays of the probes. Overexpression and purification of both Fnq26 and NphB was performed as described previously [6,9].

### Analysis by LC-MS

For further analysis the samples were applied to LC-coupled mass spectrometry (LC-MS) using a Nucleosil 100 C_18_ column (100 × 2 mm, 3 μm) and an electron spray ionisation (ESI) mass spectrometer (LC/MSD Ultra Trap System XCT 6330; Agilent 1200 series; Agilent Technology). Chromatography was carried out at a flow rate of 0.4 mL min^-1^ with a linear gradient from 10 to 100% of solvent B in 15 min (solvent A: water with 0.1% formic acid; solvent B: acetonitrile with 0.06% (v/v) formic acid). Absorbance was measured at 230, 260, 280, 360 and 435 nm. For MS analysis electron spray ionization (positive and negative ionization) in Ultra Scan mode with a capillary voltage of 3.5 kV and heated capillary temperature of 350°C was used.

## Results and Discussion

### Analysis of the genome of *Streptomyces* sp. CNQ-509

To gain insights into the biosynthesis of the hybrid terpenoids from *Streptomyces* sp. CNQ-509 we sequenced the genomic DNA and obtained a draft genome sequence. For this purpose we employed the 454-technology from Roche Applied Science on both, a paired-end and a whole-genome shotgun library and Illumina MiSeq (Nextera WGS and TruSeq PCR-free) sequencing strategies. The total of 3,978,619 reads from these data sets was assembled to a 7,998,446 nucleotide draft sequence with an average genome coverage of 87.7. The assembled draft genome consisted of 128 contigs (mean contig size 48,763 bp) and merged into seven scaffolds. Manual in silico assembly resulted in a single, linear chromosome of 8,039,333 size and an average GC content of 73.1%. Automatic functional annotation assigned 6,412 coding sequences as well as 84 tRNA and five rRNA operons. The genome sequence was deposited in NCBI under the accession no. CP011492 [29].

By using the antiSMASH 2.0 platform [40], 31 putative secondary metabolite biosynthetic gene clusters could be predicted: three siderophore, one phenazine, seven terpene, one lantipeptide, one thiopeptide-lantipeptide, one bacteriocin, one ectoine, three nonribosomal peptide synthetase (NRPS), six polyketide synthetase (two type-I-PKS, two type-II-PKS, two type-III-PKS), and three mixed polyketide synthetase gene clusters as well as two hybrid NRPS/PKS and two unspecified clusters.

### *In silico* studies

We searched the sequence for ABBA prenyltransferases via local BLAST search and identified eight genes coding for putative homologues (*AA958_30735*, *AA958_24325*, *AA958_24270*, *AA958_18620*, *AA958_12645*, *AA958_12635*, *AA958_12625* and *AA958_07645*). The gene products CnqP1 to CnqP7 show similarity to the phenol / phenazine group of prenyltransferases. CnqP8 (*AA958_07645*) is assigned to the group of bacterial indole prenyltransferases.

To gain a better understanding of their role in secondary metabolism we analysed the genetic environment of the genes for the seven putative phenol / phenazine prenyltransferases CnqP1 –CnqP7. *AA958_30735* (CnqP1) is clustered with genes characteristic for the biosynthesis of phenazine-1-carboxylic acid, homologous to the *phzABCDEFG* operon commonly found in *Pseudomonas* species [42]. *AA958_18620* (CnqP2) is located in a putative terpene biosynthesis cluster including candidate genes for a polyprenyl synthetase, a 4-hydroxy-3-methylbut-2-enyl diphosphate reductase and a ubiquinone methyltransferase. The genes *AA958_24325* (CnqP3) and *AA958_24270* (CnqP4) are part of a putative gene cluster for the biosynthesis of a hybrid polyketide-terpenoid. The cluster features genes for the formation of a naphthoquinone polyketide core i.e. putative genes for a tetrahydroxynaphthalene (THN) synthase, a THN monooxygenase and a methyltransferase as well as two putative vanadium-dependent chloroperoxidase genes. A similar genetic repertoire can be found in a gene cluster for the biosynthesis of the complex meroterpenoid antibiotics merochlorins [43,44].

The genetic context of the putative ABBA prenyltransferases CnqP5, CnqP6 and CnqP7 did not give any indication on their specific role in secondary metabolism. However, the colocation of *AA958_12625* (CnqP5), *AA958_12635* (CnqP6) and *AA958_12645* (CnqP7) with a number of putative regulators, oxidoreductases and transporters does not rule out the biosynthesis of a so far undiscovered natural product. Notably, the presence of ABBA prenyltransferase genes outside of well-defined secondary metabolic gene clusters has so far only been observed in fungi [45].

Surprisingly, genes coding for enzymes of the mevalonate pathway, typically associated with meroterpenoid biosynthesis, could not be found in the genome of *Streptomyces* sp. CNQ-509. Apparently, the isoprenoid precursor for secondary metabolites are in this case rather produced by the methylerythritol phosphate (MEP) pathway common in most eubacteria (S1 Table).

**Fig 2:**
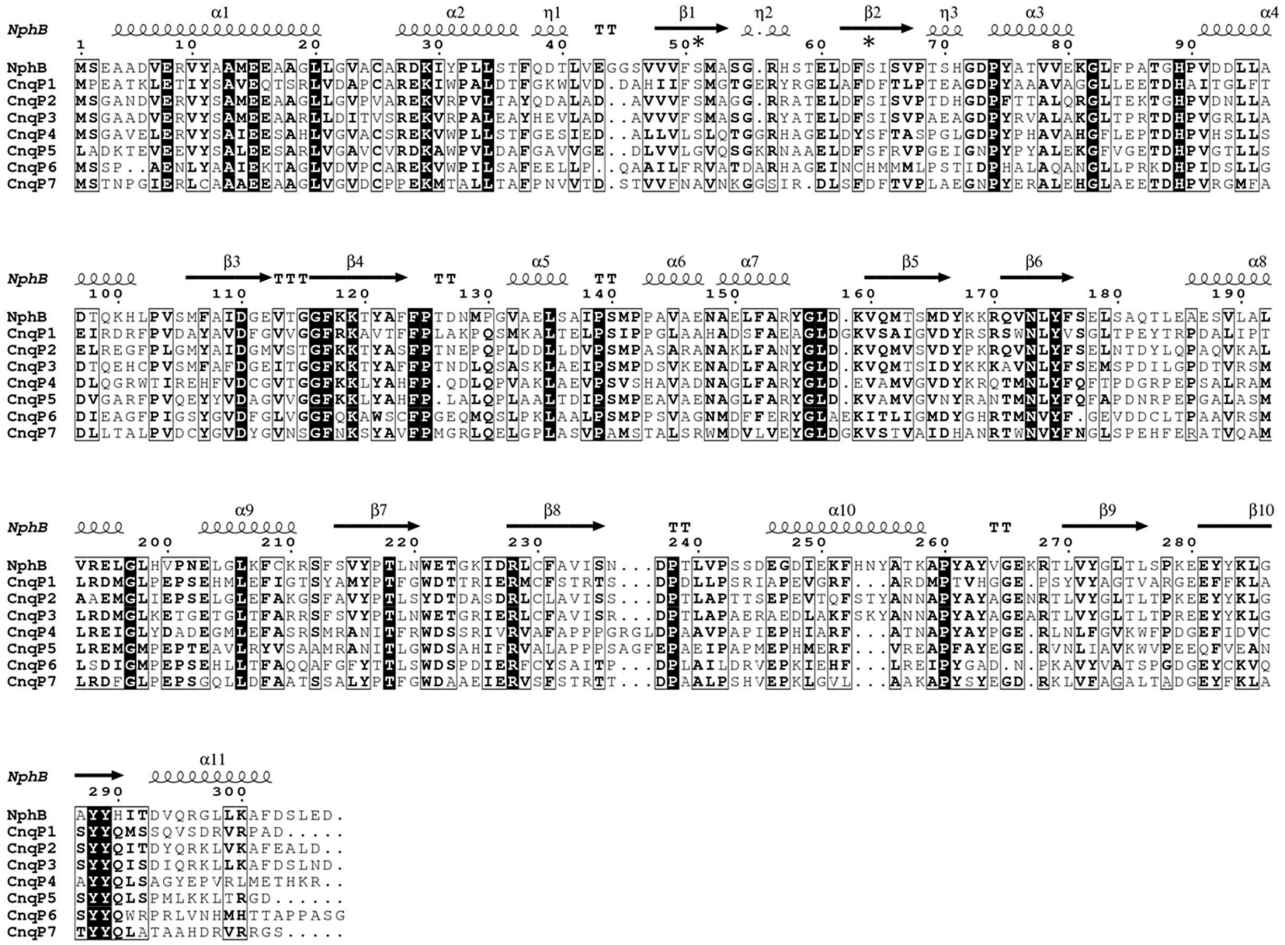
Alignment of amino acid sequences and secondary structure prediction for CnqP1 –CnqP7 visualised by ESPript. Sequence shows secondary structure elements of NphB: α, α-helices; η, 3_10_-helices; β, β-strands; TT, strict β-turns. Black box with white character for strict sequence identity, bold characters in black for similarity. * marks arginine residues typical for Mg^2+^-independent ABBA prenyltransferases.

Predictions of the three-dimensional structure of CnqP1 –CnqP7 using the Phyre^2^ server [38] revealed the characteristic PT barrel for all enzymes (data not shown). Additionally, sequence alignments and secondary structure modelling by ESPript 3.0 showed the ααββ repeat, typical for ABBA prenyltransferases (Fig 2).

**Fig 3:**
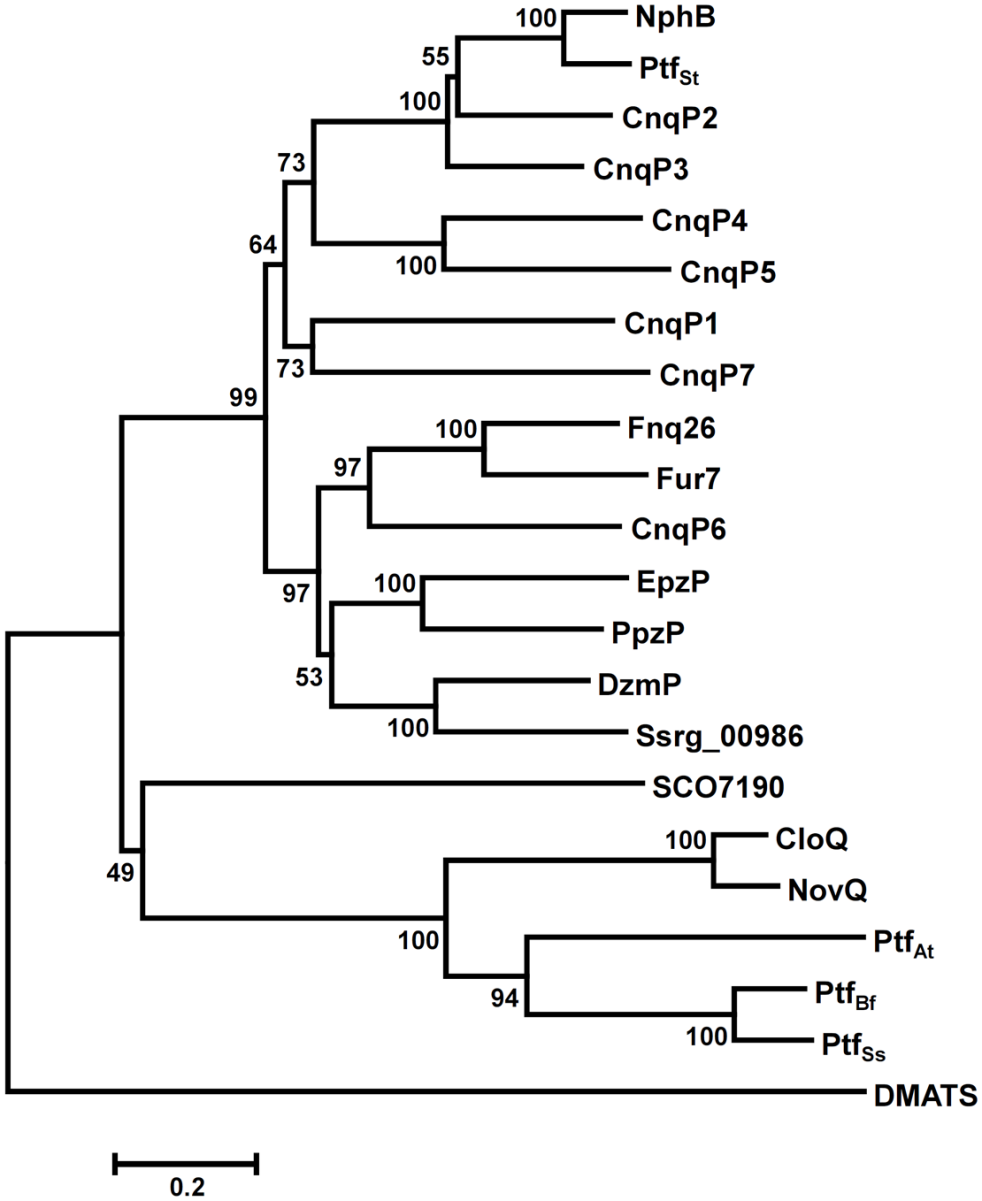
Phylogenetic tree of ABBA prenyltransferases of the phenol / phenazine family. Data include previously biochemically characterised ABBA prenyltransferases and those investigated in this study. The tree was constructed with MEGA6 using default parameter for multiple sequence alignment (CLUSTALW) and neighbour-joining method. Bootstrap values (in percent) calculated from 1000 replications are shown at the respective nodes. The fungal indole prenyltransferase DMATS (shares PT barrel) serves as a root.

To predict substrate specificity and reaction mechanism of CnqP1 –CnqP7 we then compared the amino acid sequence of these proteins with already biochemically characterised ABBA prenyltransferases of the phenol / phenazine group by construction of a phylogenetic tree (Fig 3). CnqP2 –CnqP5 were found to group with the Mg^2+^-dependent prenyltransferases NphB from *Streptomyces* sp. CL190 and Ptf_St_ from *Streptomyces tendae* [4,46]. Those are known to catalyse *C*- or *O*-prenylation of hydroxynaphthalenes as artificial aromatic substrates. CnqP6 clusters with the Mg^2+^-independent prenyltransferases Fnq26 from *Streptomyces cinnamonensis* and Fur7 from *Streptomyces* sp. strain KO-3988 [47]. These enzymes have been shown to catalyse the reverse *C*-prenylation of flaviolin. Interestingly, CnqP1 and CnqP7 form a discrete group separate from other ABBA prenyltransferases possibly indicating a different, so far not described metabolic function for these enzymes. Apparently, the high number of identified prenyltransferase genes reflects the diverse terpene chemistry from *Streptomyces* sp. CNQ-509. This prompted us to biochemically investigate the enzymatic activity of their gene products.

### Prenyltransferase activity of CnqP1 –CnqP7

#### Heterologous expression

To analyse the catalytic capacity of CnqP1 –CnqP7 *in vitro* we isolated the enzyme from recombinant *Escherichia coli*. For this purpose the putative ABBA prenyltransferases genes *AA958_30735* (CnqP1), *AA958_18620* (CnqP2), *AA958_24325* (CnqP3), *AA958_24270* (CnqP4), *AA958_12625* (CnqP5), *AA958_12635* (CnqP6) and *AA958_12645* (CnqP7) were amplified by PCR from genomic DNA and cloned into the expression vector pHis_8_. The corresponding proteins were expressed in *E*. *coli* as N-terminally His_8_-tagged proteins and purified by Ni^2+^-affinity chromatography to apparent homogeneity. Six of the enzymes were readily expressed in soluble form. Merely CnqP5 was found only in small amounts in the soluble fraction (0.6 mg/L), and additional protein concentration steps had to be carried out in order to obtain CnqP5 in sufficient concentration. SDS-PAGE gel electrophoresis of all expressed enzymes in comparison with a molecular weight standard suggests the expression of monomeric proteins (Table 1). As we previously showed that the His_8_-tag does usually not influence the activity of ABBA prenyltransferases [6], the unmodified proteins were used in the assays.

**Table 1:** Expression of CnqP1 –CnqP7.

enzyme	CnqP1	CnqP2	CnqP3	CnqP4	CnqP5	CnqP6	CnqP7
**no. aa** ^a^	300	304	305	303	299	300	299
**identity** ^b^	Fnq26 41%	Ptf_St_ 65%	NphB 65%	NphB 43%	DzmP 41%	Fnq26 53%	PpzP 38%
**calc. mass [kDa]** ^c^	33.1 / 35.2	33.2 / 35.4	33.9 / 36.1	33.5 / 35.6	32.7 / 34.9	33.0 / 35.1	32.3 / 34.4
**yield [mg L** ^**-1**^ **]** ^d^	21.1	6.9	10.4	11.2	0.6	10.6	11.5

^a^no. aa: number of amino acids;

^b^identity: best BLASTP result of a biochemically characterised ABBA prenyltransferase;

^c^calc. mass: calculated molecular mass of the genuine protein / of the octahistidyl-tagged protein;

^d^yield: purified protein from 1 L of culture

#### Substrate specificities

To investigate the substrate specificity of the purified enzymes, they were incubated with different isoprenoid and aromatic substrates. We generally used dimethylallyl diphosphate (DMAPP) and geranyl diphosphate (GPP) as isoprenoid donor molecules because farnesyl diphosphate (FPP) is rarely accepted by ABBA prenyltransferases [9]. Then again, ABBA prenyltransferases are known for their remarkable promiscuity towards aromatic substrates. To accommodate for this flexibility, we tested a diverse set of aromatic molecules: flaviolin, genistein, 1,3-dihydroxynaphthalene (1,3-DHN), 1,6-DHN, 2,7-DHN and phenazine-1-carboxylic acid (PCA) in its reduced form dihydro-PCA (S1 Fig). Moreover, 2-nitropyrrol was assayed using farnesyl diphosphate (FPP) as well as DMAPP and GPP as isoprenoid substrates. Standard conditions for the substrate screen were set as described in the section Experimental Procedures.

Only CnqP3 accepted flaviolin as a substrate. Incubation with GPP resulted in the production of a new compound distinct from 3-(3’-geranyl)-flaviolin, the main reaction product of Fnq26. MS analysis showed a molecular ion of *m/z* 341 [M-H]^-^ and thus we postulate the formation of a monogeranylated flaviolin derivative by CnqP3 (S2A and S3A Figs). Unexpectedly, flaviolin was not converted by the Fnq26 homolog CnqP6.

The isoflavonoid genistein was converted with GPP by the prenyltransferases CnqP2, CnqP3 and CnqP6. In all assays, LC-MS analysis showed a peak at *m/z* 405 [M-H]^-^, indicating the formation of a monoprenylated genistein derivative. Retention time, UV spectrum and mass matched exactly the reaction product of NphB 7-*O*-geranyl-genistein (S1, S2B–S2D and S3B Figs). Notably, CnqP3 displayed the highest product formation with 2,500 μmol_product_ mol_enzyme_^-1^ s^-1^.

1,3-DHN is an artificial substrate for ABBA prenyltransferases e.g. Fnq26 and Fur7 [6,47]. However, none of the prenyltransferases from *Streptomyces* sp. CNQ-509 showed any activity with this molecule and DMAPP or GPP.

1,6-DHN is also known to be an artificial substrate of NphB [14] and other prenyltransferases [9,32,46]. Indeed, in our investigations CnqP2, CnqP3 and CnqP5 also accepted 1,6-DHN as the aromatic precursor. Both CnqP3 and CnqP5 used DMAPP for mono-prenylation of 1,6-DHN as indicated by a single mass peak with *m/z* 227 [M-H]^-^ in LC-MS analysis of the assays (S2E–S2F and S3C Figs). GPP, on the other hand, was utilised by CnqP2 and CnqP3 to generate a new compound of *m/z* 295 [M-H]^-^ with matching HPLC-UV-MS characteristics to the NphB reaction product 5-geranyl-1,6-DHN (S1, S2G–S2H and S3D Figs). Further analysis of additional product peaks in the GPP assays with CnqP2 and CnqP3 indicate the formation of geranyl and digeranyl position isomers.

Most ABBA prenyltransferases prenylate the artificial substrate 2,7-DHN. In our substrate screen we found monogeranylated products for CnqP2, CnqP3 and CnqP4 (*m/z* 295 [M-H]^-^). For all assays retention time in HPLC and UV spectrum of the generated molecule was identical with 1-geranyl-2,7-DHN the reaction product of NphB (S1, S2I–S2K and S3E Figs). Table 2 summarises the detected product formations.

**Table 2:** Results of biochemical investigations of CnqP1 –CnqP7.

aromatic substrate	isoprenoid substrate	product formation [μmol_prod_ mol_enzyme_^-1^ s^-1^]	possible product
**flaviolin**	DMAPP	n.d.	
	GPP	CnqP3 9,800	
**genistein**	DMAPP	n.d.	
	GPP	CnqP3 2,500	7-*O*-geranylgenistein
		CnqP2 42	
		CnqP6 30	
**1,3-DHN**	DMAPP	n.d.	
	GPP	n.d.	
**1,6-DHN**	DMAPP	CnqP3 700	
		CnqP5 170	
	GPP	CnqP3 1,400	5-geranyl-1,6-DHN
		CnqP2 72	
**2,7-DHN**	DMAPP	n.d.	
		CnqP2 100	
	GPP	CnqP3 300	1-geranyl-2,7-DHN
		CnqP4 26	
**dihydro-PCA**	DMAPP	n.d.	
	GPP	n.d.	
**2-nitropyrrole**	DMAPP	n.d.	
	GPP	n.d.	
	FPP	n.d.	

Possible products are assigned by comparison of HPLC retention time as well as UV and MS spectral data of known compounds. Abbreviations: dihydroxynaphthalene (DHN), phenazine-1-carboxylic acid (PCA), dimethylallyl diphosphate (DMAPP), geranyl diphosphate (GPP), farnesyl diphosphate (FPP), not detected (n.d.).

Recently, the *O*-prenylated phenazines marinophenazine A and marinophenazine B have been isolated from *Streptomyces* sp. CNQ-509 [28]. We therefore speculated that one of the ABBA prenyltransferases might be involved in the biosynthesis of a prenylated phenazine compound and tested 5,10-dihydrophenazine-1-carboxylic acid, an established substrate of phenazine prenyltransferases [7,48], as an isoprenoid acceptor with CnqP1 –CnqP7. However, no activity was observed.

The location of CnqP1 in a phenazine biosynthetic gene cluster makes this enzyme a likely candidate for phenazine prenyltransferase activity. We thus assayed CnqP1 with six further phenazine-derived substrates: non-reduced phenazine-1-carboxylic acid, phenazine-1-carboxylic acid methyl ester, *N*-methyl-phenazine (as methyl sulphate salt), pyocyanine, phenazine-5-*N*-oxide and 1,6-dihydroxyphenazine. Contrary to our expectations, none of these molecules was accepted by the enzyme, providing no support for the hypothesis that CnqP1 is responsible for the prenylation reaction in marinophenazine biosynthesis. Finally, we could show that a completely different type of prenyltransferase, i.e. an integral membrane protein, catalyses the prenylation reaction in marinophenazine biosynthesis [32].

To gain insight into the biosynthesis of the nitropyrrolins we also tested 2-nitropyrrolin as an aromatic substrate. However, none of the prenyltransferases showed activity using 2-nitropyrrol with FPP or DMAPP or GPP. Therefore, we cloned, expressed and tested additionally the putative indole prenyltransferase CnqP8, but again could not observe any activity for 2-nitropyrrol and one of the isoprenoid substrates (data not shown).

#### Dependency on magnesium ions

Most ABBA prenyltransferases characterised up to now are independent of the presence of magnesium ions. However, when we investigated the reactions of CnqP2 (2,7-DHN and GPP), CnqP3 (genistein and GPP), CnqP4 (2,7-DHN and GPP) and CnqP6 (genistein and GPP), prenyltransferase activity was readily detected in the presence of 5 mM MgCl_2_. Omission of MgCl_2_ and addition of EDTA (1 mM) completely abolished the activity in all assays, proving the magnesium dependency of these enzymes.

## Discussion / Conclusions

*Streptomyces* sp. CNQ-509 is a remarkable producer of diverse terpenoid compounds. In this study we used a genome-based approach to increase our understanding of the mixed terpenoid metabolism of this marine derived strain. Our search for ABBA prenyltransferases yielded eight putative genes coding for prenyltransferases whereof five gene products showed biochemical activity *in vitro* with a variety of aromatic acceptor molecules. To our knowledge *Streptomyces* sp. CNQ-509 is the first reported strain that harbours ABBA prenyltransferases in such high numbers.

Of the five prenyltransferases with *in vitro* activity, CnqP3 stands out as it displayed remarkable promiscuity with respect not only to the aromatic but also to the isoprenoid substrate molecules. Flaviolin, genistein, 1,6-DHN and 2,7-DHN were readily converted by CnqP3. CnqP3 thus is a promising candidate biocatalyst for chemoenzymatic synthesis and diversification. Based on these results and gene cluster analysis we postulate that CnqP3 might be involved in debromomarinone formation in *Streptomyces* sp. CNQ-509. However, we were not able to assign any of the investigated ABBA prenyltransferases to nitropyrrolin or marinophenazine biosynthesis. As mentioned above, we eventually found that the prenylation reaction in marinophenazine biosynthesis is carried out by a completely different type of enzyme [32]. It remains to be shown whether the prenylation reaction in nitropyrrolin biosynthesis is also carried out by a different type of enzyme. Alternatively, CnqP1 or CnqP7 which differ in their amino acid sequence from previously known ABBA prenyltransferase and which showed no activity with any of the phenol/phenazine substrates tested in this study, may be responsible for the prenylation reaction in nitropyrrolin biosynthesis, but may require substrates or conditions different from the ones tested in this study.

So far, only three ABBA prenyltransferases have been found to be magnesium-dependent, i.e. NphB, Ptf_St_ and Mcl23 [3,44,46]. Our study now identifies four further magnesium-dependent ABBA prenyltransferases, indicating that Mg^2+^-dependency maybe more common among this superfamily than previously known. CnqP6 was predicted to be Mg^2+^-independent from its amino acid sequence, based on the presence of an arginine residue typical for the Mg^2+^-independent ABBA prenyltransferases (Fig 2). Surprisingly, biochemical investigation clearly showed the magnesium dependency of CnqP6. This observation may warrant further investigations in order to identify the structural basis for the different cofactor requirement of CnqP6 and NphB.

Mixed-terpenoid compounds are of extraordinary interest in the search for new biologically active products. Different approaches to screen the increasing number of sequenced bacterial genomes for the potential of the organisms to form promising compounds have been reported. One of these strategies is searching for the presence of the *hmgr* gene, coding for a key enzyme of the mevalonate pathway [49]. Actinomycetes normally utilise the methylerythritol phosphate pathway to produce isoprenoids of the primary metabolism, though examples for the involvement in secondary metabolism have been reported [50]. In contrast, the presence of the mevalonate pathway is restricted to strains which produce isoprenoid secondary metabolites [51]. Hydroxymethylglutaryl-CoA reductase (*hmgr*) is a key enzyme in the mevalonate pathway and has been successfully employed to identify actinomycete strains capable of producing new isoprenoid secondary metabolites [48,52]. However, our study now shows the absence of *hmgr* or other genes of the mevalonate pathway in the genome of *Streptomyces* sp. CNQ-509, a particularly rich producer of hybrid isoprenoid compounds. Hence, we propose that screening for the presence of genes for ABBA prenyltransferases may be useful to complement screenings for the presence of *hmgr* in the identification of actinomycete strains producing new mixed-terpenoid secondary metabolites.

## Supporting Information

S1 Fig Aromatic substrates used for prenyltransferase assays and predicted products. (PDF)

S2 Fig HPLC-UV analysis of prenyltransferase assays A–K. Upper chromatograms: assay without enzyme. Lower chromatograms: assay with indicated enzyme. The main product is highlighted with an arrow. **A** reaction of CnqP3, flaviolin and GPP; **B** reaction of CnqP2, genistein and GPP; **C** reaction of CnqP3, genistein and GPP; **D** reaction of CnqP6, genistein and GPP; **E** reaction of CnqP3, 1,6-DHN and DMAPP; **F** reaction of CnqP5, 1,6-DHN and DMAPP; **G** reaction of CnqP2, 1,6-DHN and GPP; **H** reaction of CnqP3, 1,6-DHN and GPP; **I** reaction of CnqP2, 2,7-DHN and GPP; **J** reaction of CnqP3, 2,7-DHN and GPP; **K** reaction of CnqP4, 2,7-DHN and GPP.
(PDF)

S3 Fig MS analysis of enzymatic products. Extracted ion chromatograms (EICs) and MS/MS spectra. **A** Reaction of flaviolin: Monoprenylated product with GPP, *m/z* 341.1 [M-H]^-^. **B** Reaction of genistein: Monoprenylated product with GPP, *m/z* 405.0 [M-H]^-^. **C** Reaction of 1,6-dihydroxy naphthalene (1,6-DHN): Monoprenylated product with DMAPP, *m/z* 227.0 [M-H]^-^. **D** Reaction of 1,6-dihydroxynaphthalene (1,6-DHN): Monoprenylated product with GPP, *m/z* 295.0 [M-H]^-^. **E** Reaction of 2,7-dihydroxynaphthalene (2,7-DHN): Monoprenylated product with GPP, *m/z* 295.0 [M-H]^-^.
(PDF)

S1 Table Homologues of the methylerythritol phosphate (MEP) pathway. (PDF)
