# Supplementary figures and images for: Diversity of ABBA Prenyltransferases in Marine Streptomyces sp. CNQ-509: Promiscuous Enzymes for the Biosynthesis of Mixed Terpenoid Compounds
Source: PLoS One. 2015 Dec 14;10(12):e0143237. doi: 10.1371/journal.pone.0143237 (PMC4684245; doi:10.1371/journal.pone.0143237)

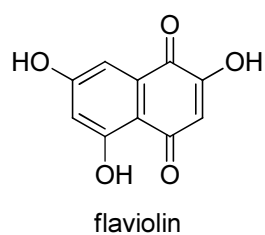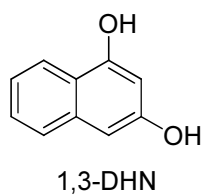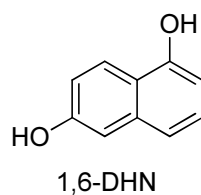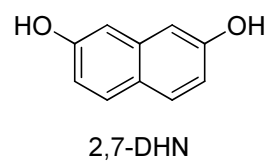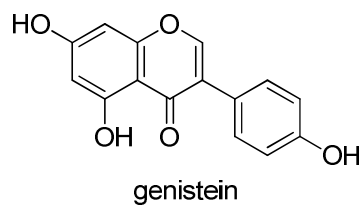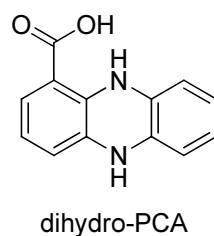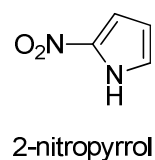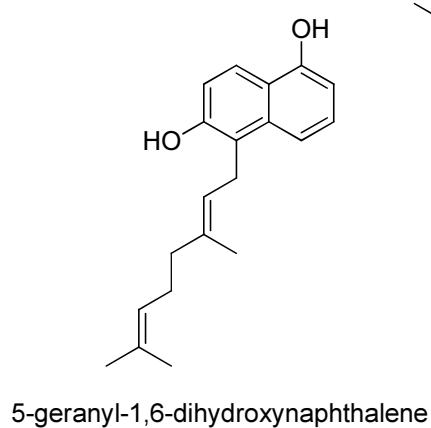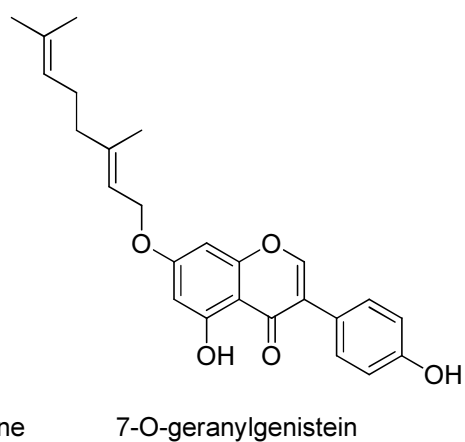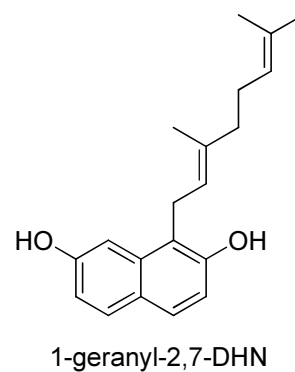

**S1 Fig.** Aromatic substrates used for prenyltransferase assays and predicted products.

Supplement: S1 Fig — (PDF) [file pone.0143237.s001.pdf]
