# Supplementary material for: Diversity of ABBA Prenyltransferases in Marine Streptomyces sp. CNQ-509: Promiscuous Enzymes for the Biosynthesis of Mixed Terpenoid Compounds
Source: PLoS One. 2015 Dec 14;10(12):e0143237. doi: 10.1371/journal.pone.0143237 (PMC4684245; doi:10.1371/journal.pone.0143237)
Supplement: S2 Fig — Upper chromatograms: assay without enzyme. Lower chromatograms: assay with indicated enzyme. The main product is highlighted with an arrow. A reaction of CnqP3, flaviolin and GPP; B reaction of CnqP2, genistein and GPP; C reaction of CnqP3, genistein and GPP; D reaction of CnqP6, genistein and GPP; E reaction of CnqP3, 1,6-DHN and DMAPP; F reaction of CnqP5, 1,6-DHN and DMAPP; G reaction of CnqP2, 1,6-DHN and GPP; H reaction of CnqP3, 1,6-DHN and GPP; I reaction of CnqP2, 2,7-DHN and GPP; J reaction of CnqP3, 2,7-DHN and GPP; K reaction of CnqP4, 2,7-DHN and GPP. (PDF) [file pone.0143237.s002.pdf]

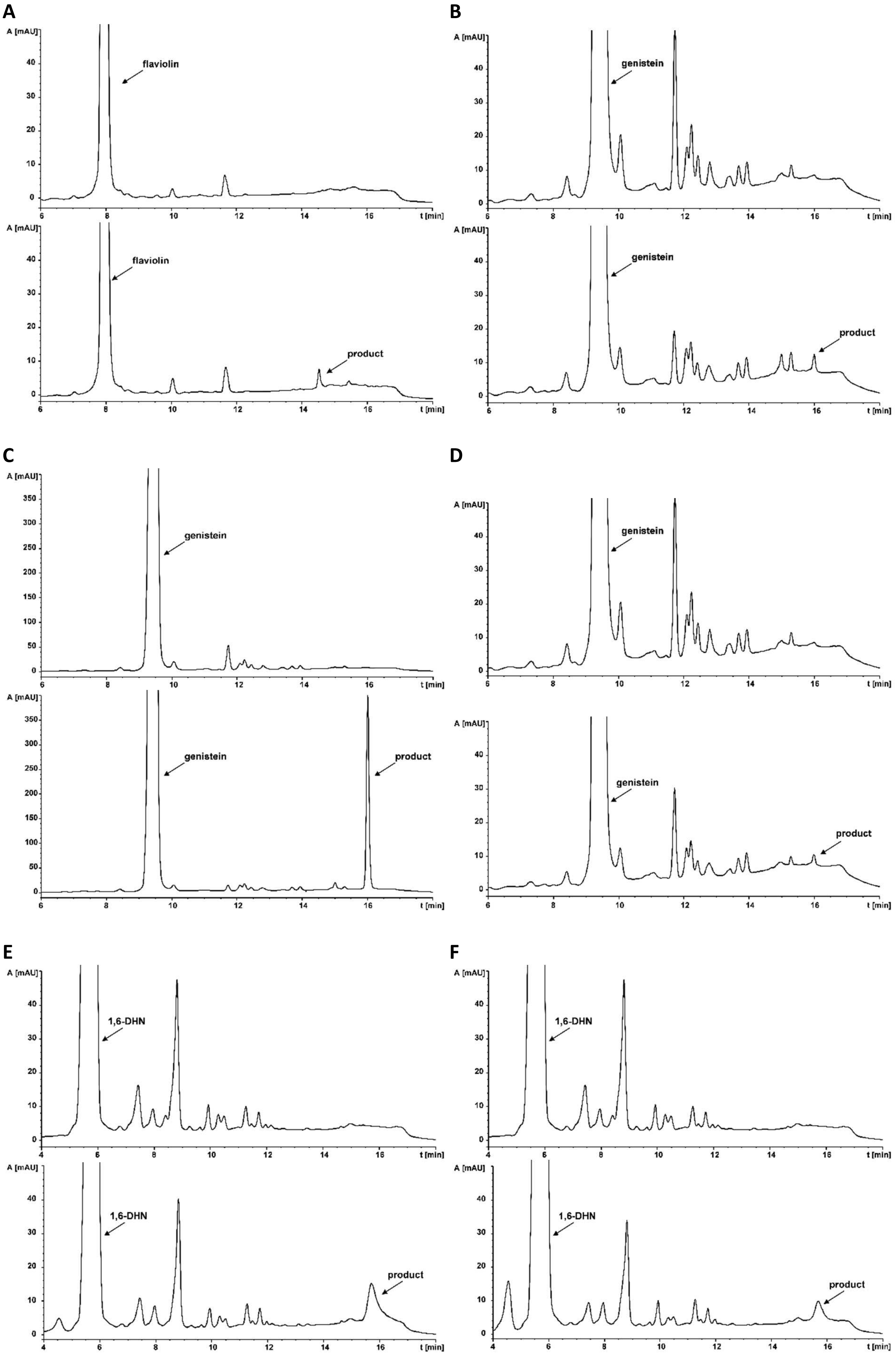

A
B
C
D
E
F
A [mAU]
t [min]
flaviolin
genistein
1,6-DHN
product

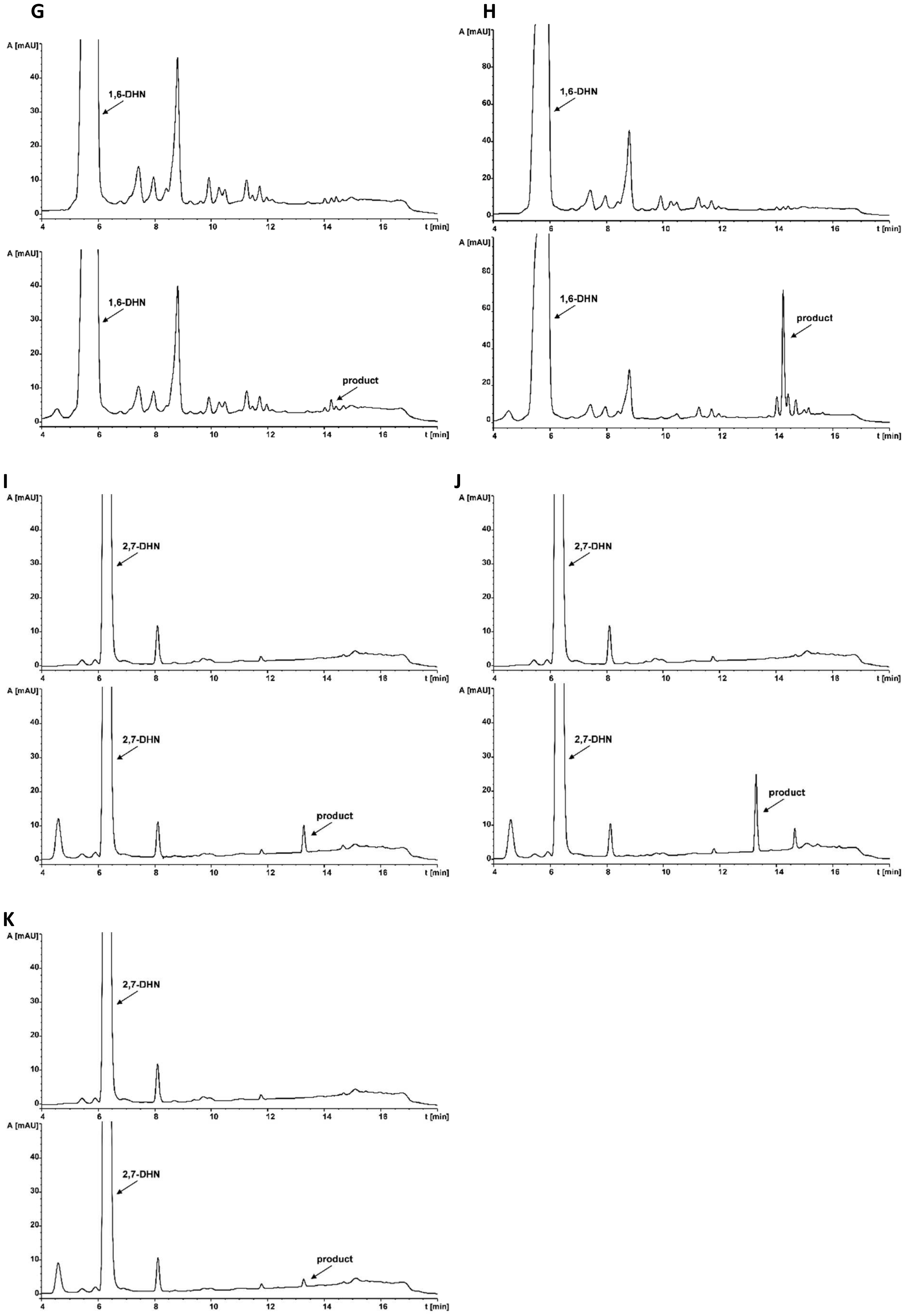

G
A [mAU]
1,6-DHN
t [min]
A [mAU]
1,6-DHN
product
t [min]
H
A [mAU]
1,6-DHN
t [min]
A [mAU]
1,6-DHN
product
t [min]
I
A [mAU]
2,7-DHN
t [min]
A [mAU]
2,7-DHN
product
t [min]
J
A [mAU]
2,7-DHN
t [min]
A [mAU]
2,7-DHN
product
t [min]
K
A [mAU]
2,7-DHN
t [min]
A [mAU]
2,7-DHN
product
t [min]

S2 Fig. HPLC-UV analysis of prenyltransferase assays **A** – **K**. Upper chromatograms: assay without enzyme. Lower chromatograms: assay with indicated enzyme. The main product is highlighted with an arrow. **A** reaction of CnqP3, flaviolin and GPP; **B** reaction of CnqP2, genistein and GPP; **C** reaction of CnqP3, genistein and GPP; **D** reaction of CnqP6, genistein and GPP; **E** reaction of CnqP3, 1,6-DHN and DMAPP; **F** reaction of CnqP5, 1,6-DHN and DMAPP; **G** reaction of CnqP2, 1,6-DHN and GPP; **H** reaction of CnqP3, 1,6-DHN and GPP; **I** reaction of CnqP2, 2,7-DHN and GPP; **J** reaction of CnqP3, 2,7-DHN and GPP; **K** reaction of CnqP4, 2,7-DHN and GPP.
