# Supplementary material for: Diversity of ABBA Prenyltransferases in Marine Streptomyces sp. CNQ-509: Promiscuous Enzymes for the Biosynthesis of Mixed Terpenoid Compounds
Source: PLoS One. 2015 Dec 14;10(12):e0143237. doi: 10.1371/journal.pone.0143237 (PMC4684245; doi:10.1371/journal.pone.0143237)
Supplement: S3 Fig — Extracted ion chromatograms (EICs) and MS/MS spectra. A Reaction of flaviolin: Monoprenylated product with GPP, m/z 341.1 [M-H]-. B Reaction of genistein: Monoprenylated product with GPP, m/z 405.0 [M-H]-. C Reaction of 1,6-dihydroxy naphthalene (1,6-DHN): Monoprenylated product with DMAPP, m/z 227.0 [M-H]-. D Reaction of 1,6-dihydroxynaphthalene (1,6-DHN): Monoprenylated product with GPP, m/z 295.0 [M-H]-. E Reaction of 2,7-dihydroxynaphthalene (2,7-DHN): Monoprenylated product with GPP, m/z 295.0 [M-H]-. (PDF) [file pone.0143237.s003.pdf]

A

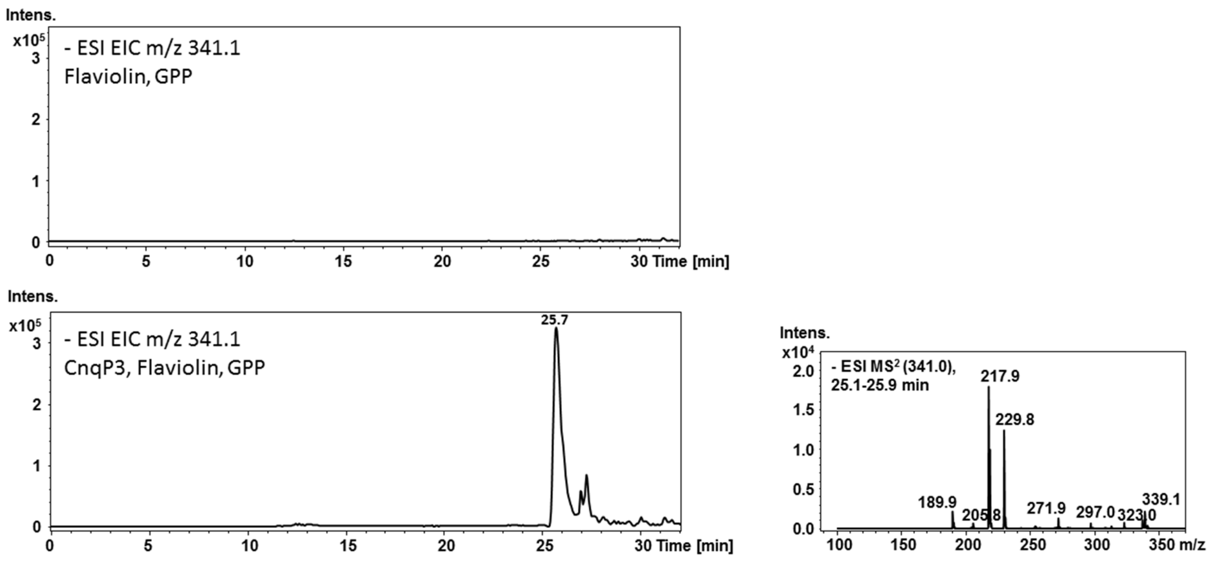

Intens.
x10^5
- ESI EIC m/z 341.1
Flaviolin, GPP
Time [min]
Intens.
x10^5
25.7
- ESI EIC m/z 341.1
CnqP3, Flaviolin, GPP
Time [min]
Intens.
x10^4
- ESI MS2 (341.0),
25.1-25.9 min
217.9
229.8
189.9
205.8
271.9
297.0
323.0
339.1
m/z

B

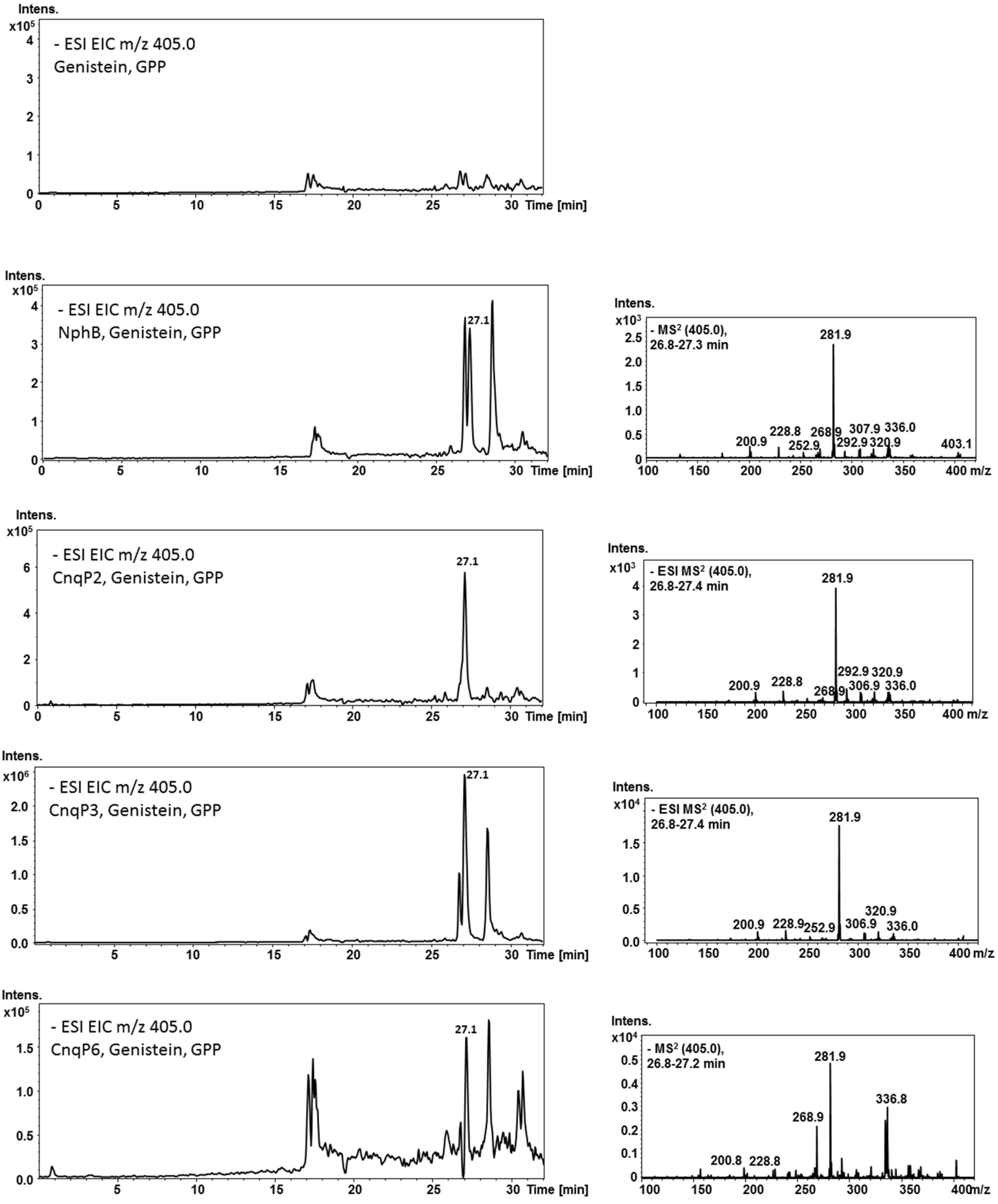

Intens.
- ESI EIC m/z 405.0
Genistein, GPP
Time [min]
- ESI EIC m/z 405.0
NphB, Genistein, GPP
27.1
- MS2 (405.0), 26.8-27.3 min
281.9
200.9
228.8
252.9
268.9
292.9
307.9
320.9
336.0
403.1
m/z
- ESI EIC m/z 405.0
CnqP2, Genistein, GPP
27.1
- ESI MS2 (405.0), 26.8-27.4 min
281.9
200.9
228.8
268.9
292.9
306.9
320.9
336.0
- ESI EIC m/z 405.0
CnqP3, Genistein, GPP
27.1
- ESI MS2 (405.0), 26.8-27.4 min
281.9
200.9
228.9
252.9
306.9
320.9
336.0
- ESI EIC m/z 405.0
CnqP6, Genistein, GPP
27.1
- MS2 (405.0), 26.8-27.2 min
281.9
268.9
336.8
200.8
228.8

C

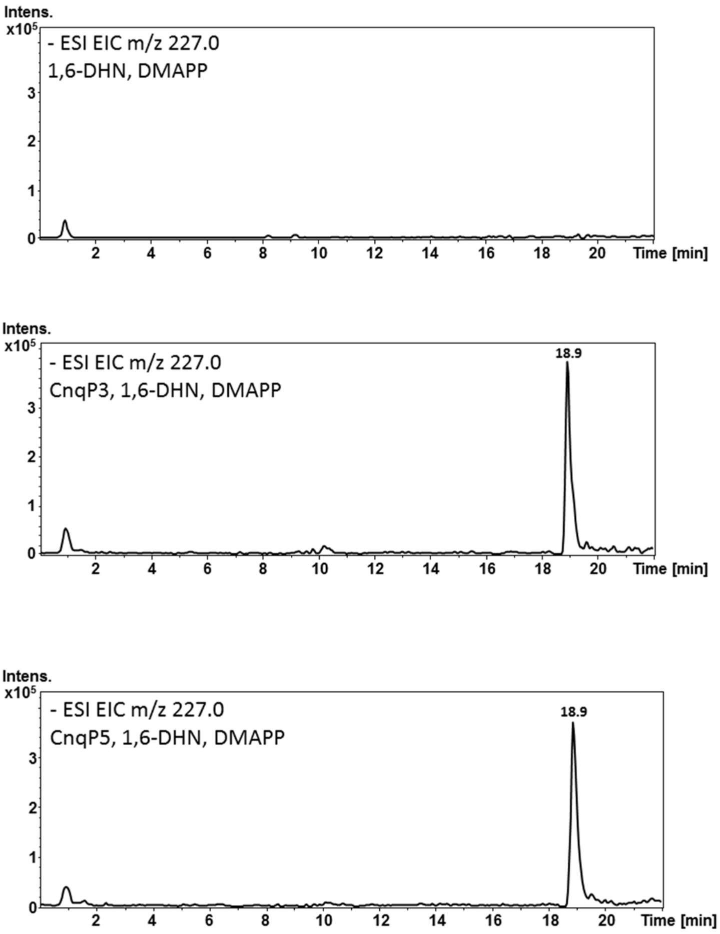
Intens.
x10^5
- ESI EIC m/z 227.0
1,6-DHN, DMAPP
0
1
2
3
2
4
6
8
10
12
14
16
18
20
Time [min]
Intens.
x10^5
- ESI EIC m/z 227.0
CnqP3, 1,6-DHN, DMAPP
18.9
0
1
2
3
2
4
6
8
10
12
14
16
18
20
Time [min]
Intens.
x10^5
- ESI EIC m/z 227.0
CnqP5, 1,6-DHN, DMAPP
18.9
0
1
2
3
2
4
6
8
10
12
14
16
18
20
Time [min]

D

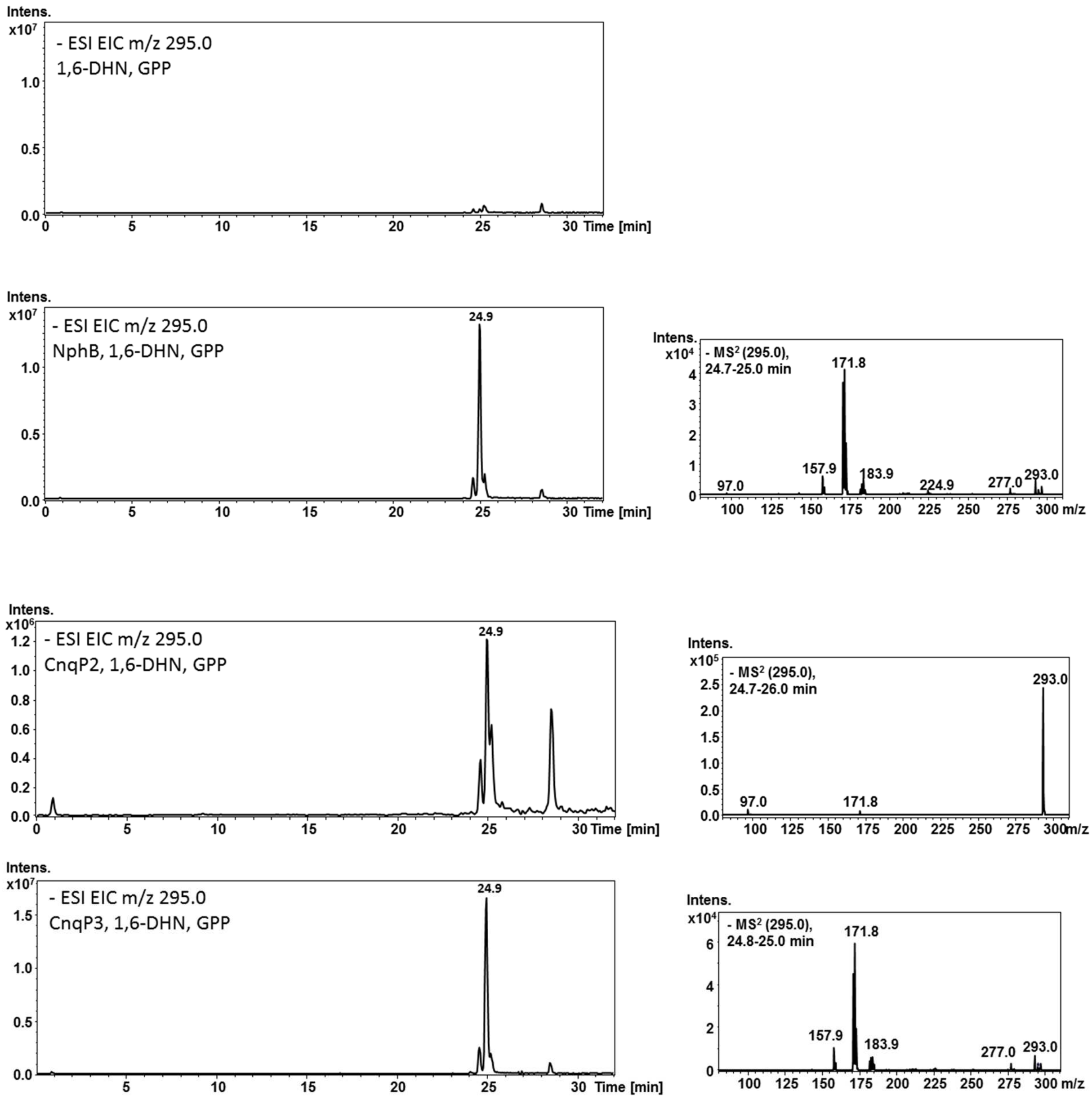
Intens.
x10^7
- ESI EIC m/z 295.0
1,6-DHN, GPP
1.0
0.5
0.0
0 5 10 15 20 25 30 Time [min]
Intens.
x10^7
- ESI EIC m/z 295.0
NphB, 1,6-DHN, GPP
24.9
1.0
0.5
0.0
0 5 10 15 20 25 30 Time [min]
Intens.
x10^4
- MS² (295.0),
24.7-25.0 min
171.8
157.9
183.9
97.0
224.9
277.0
293.0
4 3 2 1 0
100 125 150 175 200 225 250 275 300 m/z
Intens.
x10^6
- ESI EIC m/z 295.0
CnqP2, 1,6-DHN, GPP
24.9
1.2 1.0 0.8 0.6 0.4 0.2 0.0
0 5 10 15 20 25 30 Time [min]
Intens.
x10^5
- MS² (295.0),
24.7-26.0 min
293.0
97.0
171.8
2.5 2.0 1.5 1.0 0.5 0.0
100 125 150 175 200 225 250 275 300 m/z
Intens.
x10^7
- ESI EIC m/z 295.0
CnqP3, 1,6-DHN, GPP
24.9
1.5 1.0 0.5 0.0
5 10 15 20 25 30 Time [min]
Intens.
x10^4
- MS² (295.0),
24.8-25.0 min
171.8
157.9
183.9
277.0
293.0
6 4 2 0
100 125 150 175 200 225 250 275 300 m/z

E

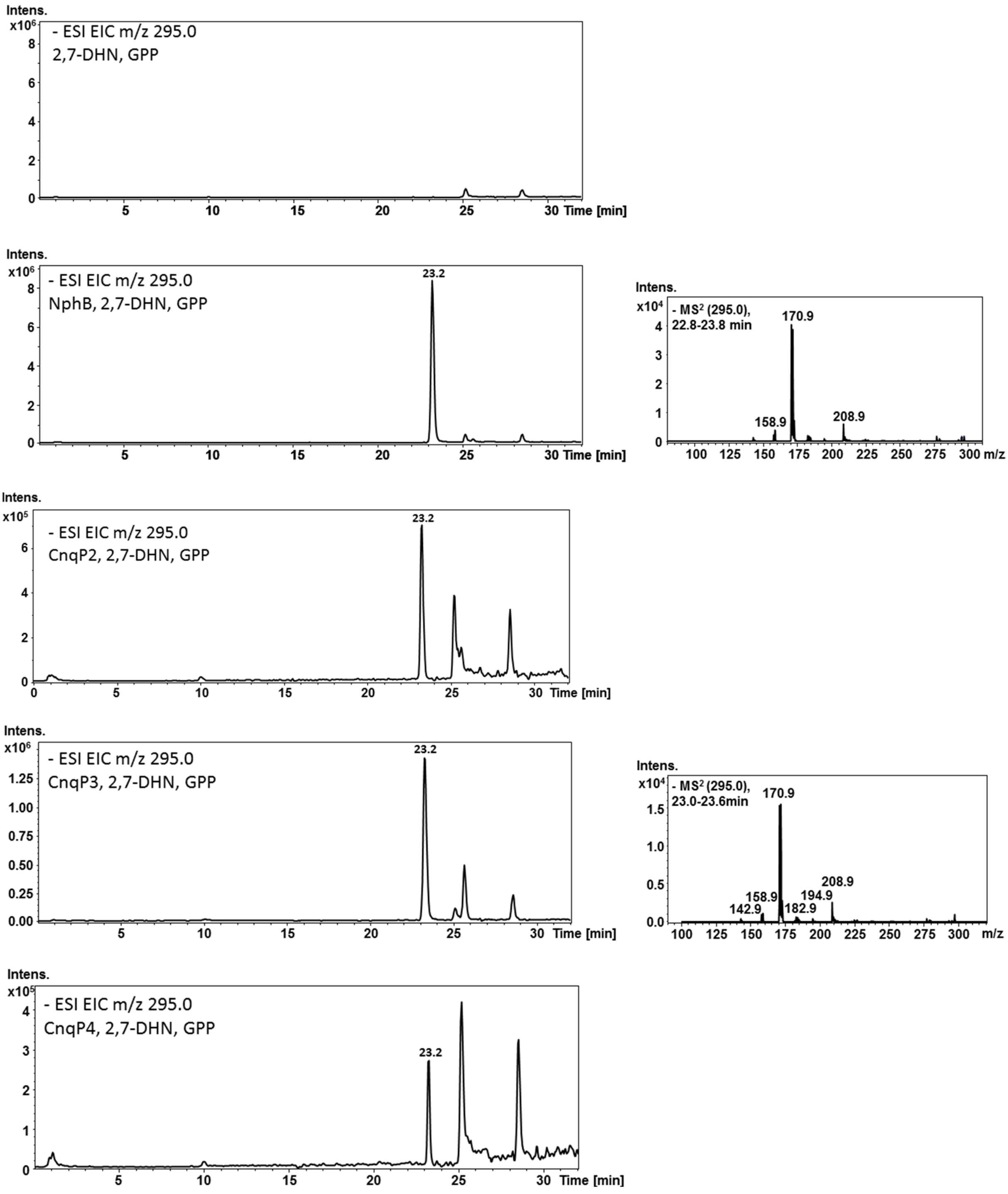

S3 Fig. MS analysis of enzymatic products. Extracted ion chromatograms (EICs) and MS/MS spectra. **A** Reaction of flaviolin: Monoprenylated product with GPP, *m/z* 341.1 $[M-H]^-$. **B** Reaction of genistein: Monoprenylated product with GPP, *m/z* 405.0 $[M-H]^-$. **C** Reaction of 1,6-dihydroxy naphthalene (1,6-DHN): Monoprenylated product with DMAPP, *m/z* 227.0 $[M-H]^-$. **D** Reaction of 1,6-dihydroxynaphthalene (1,6-DHN): Monoprenylated product with GPP, *m/z* 295.0 $[M-H]^-$. **E** Reaction of 2,7-dihydroxynaphthalene (2,7-DHN): Monoprenylated product with GPP, *m/z* 295.0 $[M-H]^-$.
