# Supplementary material for: Diversity of ABBA Prenyltransferases in Marine Streptomyces sp. CNQ-509: Promiscuous Enzymes for the Biosynthesis of Mixed Terpenoid Compounds
Source: PLoS One. 2015 Dec 14;10(12):e0143237. doi: 10.1371/journal.pone.0143237 (PMC4684245; doi:10.1371/journal.pone.0143237)
Supplement: S1 Table — (PDF) [file pone.0143237.s004.pdf]

S1 Table. Homologues of the methylerythritol phosphate (MEP) pathway

Gene	AA	Protein homologue	Accession number	Identity / similarity	Proposed function
AA958_ 27820	655	B105_RS0102225, *Streptomyces* sp. CNT371	WP_027740976	95 / 97	deoxyxylulose phosphate (DXP) synthase
AA958_ 24380	635	B121_RS0122075, *Streptomyces* sp. CNH099	WP_027755386	93 / 96	
AA958_ 26245	419	B105_RS0118250, *Streptomyces* sp. CNT371	WP_027743595	97 / 98	DXP reducto isomerase
AA958_ 13460	269	B121_RS0109340, *Streptomyces* sp. CNH099	WP_027753224	96 / 98	MEP-cytidyl transferase
AA958_ 12100	321	I003_RS0122715, *Streptomyces* sp. CNQ329	WP_027773621	88 / 91	diphosphocytidyl-ME kinase
AA958_1 3455	178	B121_RS0109345, *Streptomyces* sp. CNH099	WP_027753225	94 / 98	ME cyclodiphosphate synthase
AA958_ 26255	384	B121_RS0119255, *Streptomyces* sp. CNH099	WP_027754914	98 / 98	hydroxymethyl-butenyl diphosphate (HMBDP) synthase
AA958_ 24310	382	B121_RS0131790, *Streptomyces* sp. CNH099	WP_027756924	95 / 98	
AA958_ 22700	305	I003_RS0110900, *Streptomyces* sp. CNQ329	WP_027771554	96 / 98	HMBDP reductase
AA958_ 24320	312	IO24_RS0138155, *Streptomyces* flavovariabilis	WP_031144193	80 / 91	HMBDP reductase
AA958_ 18615	311	IO24_RS0138155, *Streptomyces* flavovariabilis	WP_031144193	78 / 87	HMBDP reductase
